# Supplementary material for: A fungal substrate mimicking molecule suppresses plant immunity via an inter-kingdom conserved motif
Source: Nat Commun. 2019 Apr 5;10:1576. doi: 10.1038/s41467-019-09472-8 (PMC6450895; doi:10.1038/s41467-019-09472-8)
Supplement: Supplementary file 1 — Supplementary Information File [file 41467_2019_9472_MOESM1_ESM.pdf]

Supplementary Information

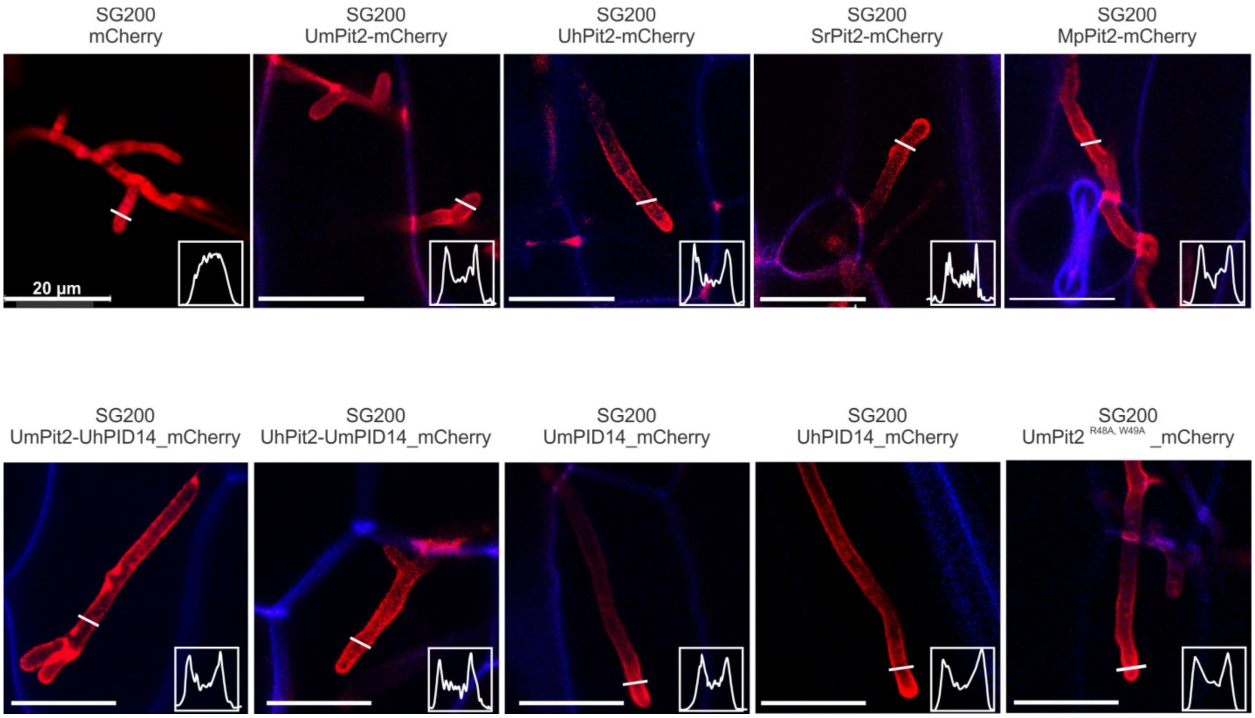

Supplementary Figure 1. Localization of mCherry tagged proteins in SG200. SG200 was transformed with C-terminal tagged mCherry Pit2 proteins from *U. maydis* (UmPit2), *S. reilianum* (SrPit2), *U. hordei* (UhPit2), *M. pennsylvanicum* (MpPit2) and the *U. maydis* mutant R48AW49A (UmPit2 ^{R48AW49A}) as well as with the chimeras UmPit2-UhPID14 and UhPit2-UmPID14 and the PID14 peptides from *U. maydis* (UmPID14) and *U. hordei* (UhPID14). All constructs were expressed under the native *pit2* promotor without introns and carrying the *pit2* signal peptide. As a control a mCherry construct without signal peptide was also included. Early Golden Bantam (EGB) maize seedlings were infiltrated and after 2 days samples were analyzed under confocal microscope. Fluorescent quantification was performed in a cross section of the hyphae (white line) and plotted on the right side of each image (graph in the box). The mCherry construct shows fluorescence in the inner part of the hyphae, indicating cytoplasmic localization whereas all other constructs show two peaks of fluorescence surrounded the hyphal membrane suggesting apoplastic localization. Scale bar: 20 µM.

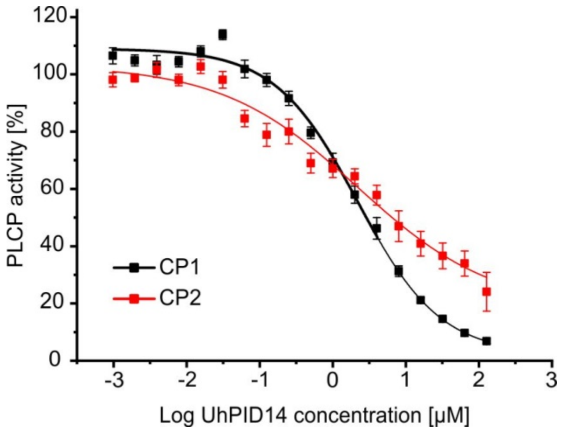

Supplementary Figure 2. UhPID14 inhibitory profile of *N. benthamiana* over-expressed CP1 and CP2. Maize CP1 (CP1a) and CP2 proteases were overexpressed in *N. benthamiana* leaves via agrobacterium mediated transformation. Apoplastic fluids were isolated and tested for PLCP activity using the probe MV201 [1]. Activity was normalized based on activity blots. Apoplastic fluids containing active CP1 and CP2 were pre-incubated for 5 min with increasing concentrations of heterologous expressed UhPID14 (0 – 128 µM) and PLCP activity was monitored using the fluorogenic substrate Z-Leu-Arg-AMC . PLCP activity was normalized using the non - inhibitor control and set to 100 %. Values are means of at least three independent biological replicates and the error bars represent the standard error of the mean (SEM).

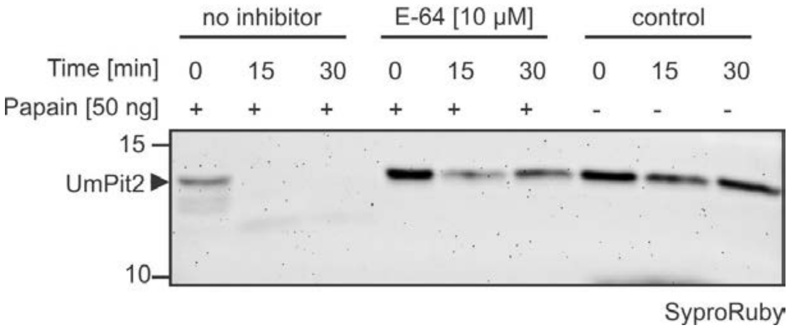

Supplementary Figure 3. Papain degrades UmPit2 over time. Commercially available papain was incubated with 2 µg heterologous expressed UmPit2 and degradation was monitored over time (0, 15 and 30 min). As a negative control, papain was 15 min pre-incubated with 10 µM E-64, an inhibitor of papain activity, before the addition of UmPit2. The control samples are incubations of UmPit2 over time without the addition of papain. Samples were loaded on SDS gels and stained with SyproRuby. This experiment has been repeated three times with similar results.

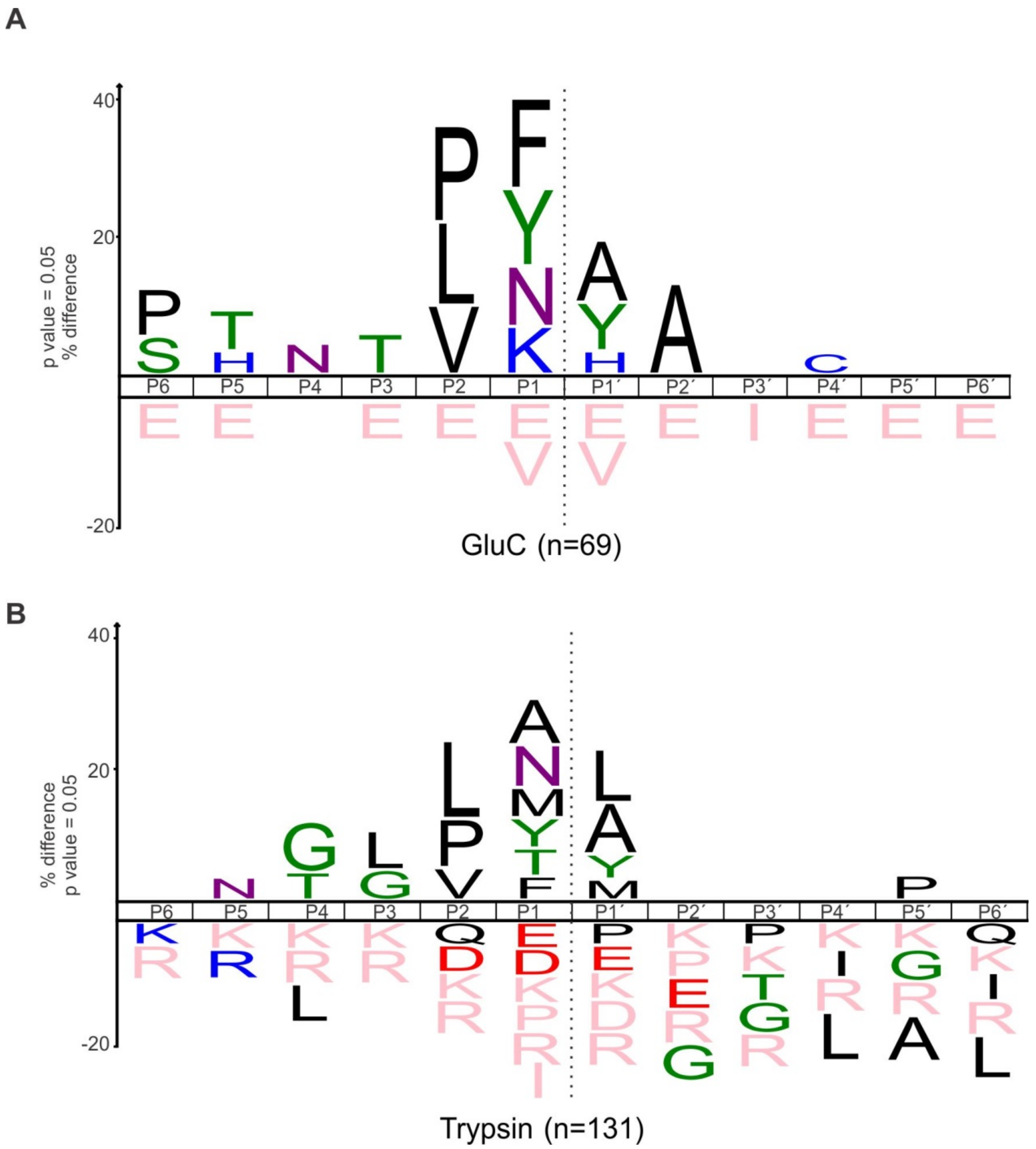

Supplementary Figure 4. Substrate specificity of PLCPs in F-24 using PICS. F-24 was pre-incubated for 10 min with a mix of inhibitors (1mM EDTA, 50 µM Pepstatin A and 50 µM DCI) to avoid protease activity from other proteases different than PLCPs. GluC- **(A)** or tryptic- **(B)** generated peptide libraries were incubated with previously treated F-24 at protease-to-library ratios of 1:100 for 16h at room temperature. Reaction was stopped with 3 M guanidine final volume and samples were then subjected to PICS analysis. Semi-specific peptides with at least four-fold increase in intensity upon F-24 addition were used for reconstruction of the cleavage sites displayed as IceLogos. Amino acids are abbreviated using their one letter code. P: substrate position non-prime site; P’: substrate position prime site. The dashed line represents the cleavage site.

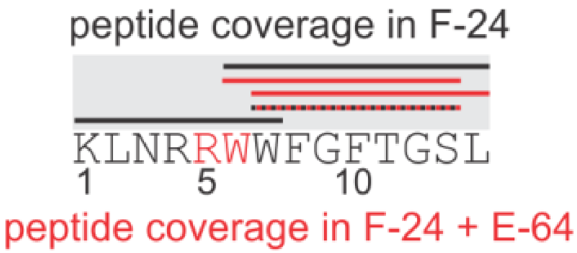

Supplementary Figure 5. Processing of UmPID14 in F-24. F-24 was pre-incubated for 10 min with 10 µM E-64 or with inhibitor mix containing E-64. 20 µM UmPID14 was added to pre-treated fractions and further incubated at room temperature for 30 min. Reaction was stopped by addition of 3 M guanidine final volume and subjected to MS analysis. Shown is the peptide coverage in F-24 represented as black lines and in F-24 with E-64 represented as red lines. Shared peptides between both samples are shown in dashed red-black lines.

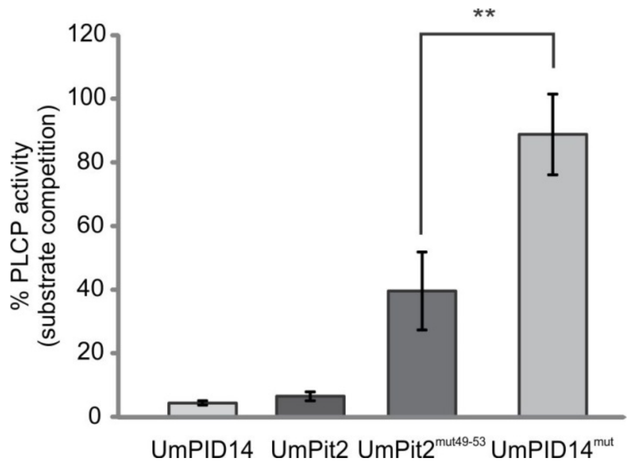

Supplementary Figure 6. UmPit2 rather than UmPID14 is preferentially used as substrate. The activity of maize apoplastic fluids was tested using the fluorogenic substrate Z-Phe-Arg-AMC. A competition experiment was performed by incubating SA treated apoplastic fluids with 10 µM substrate (Z-Phe-Arg-AMC) and same molar concentrations for UmPit2, UmPit2$^{mut49-53}$, were PID14 is mutated to W49G, W50G, F51G, and F53G [2], UmPID14 and UmPID14^{mut} carrying the same mutations in the PID14 as UmPit2$^{mut49-53}$ [2]. UmPit2 as well as UmPID14 mutants cannot inhibit maize apoplastic PLCPs [2]. Activity was set to 100 % in the presence of buffer for proteins or water for peptides. Values are means of three independent biological replicates and error bars represent standard deviation (SD). Starts show significant differences between UmPit2$^{mut49-53}$ and UmPID14^{mut} ($p=0.0084$ **) using a t-test analysis.

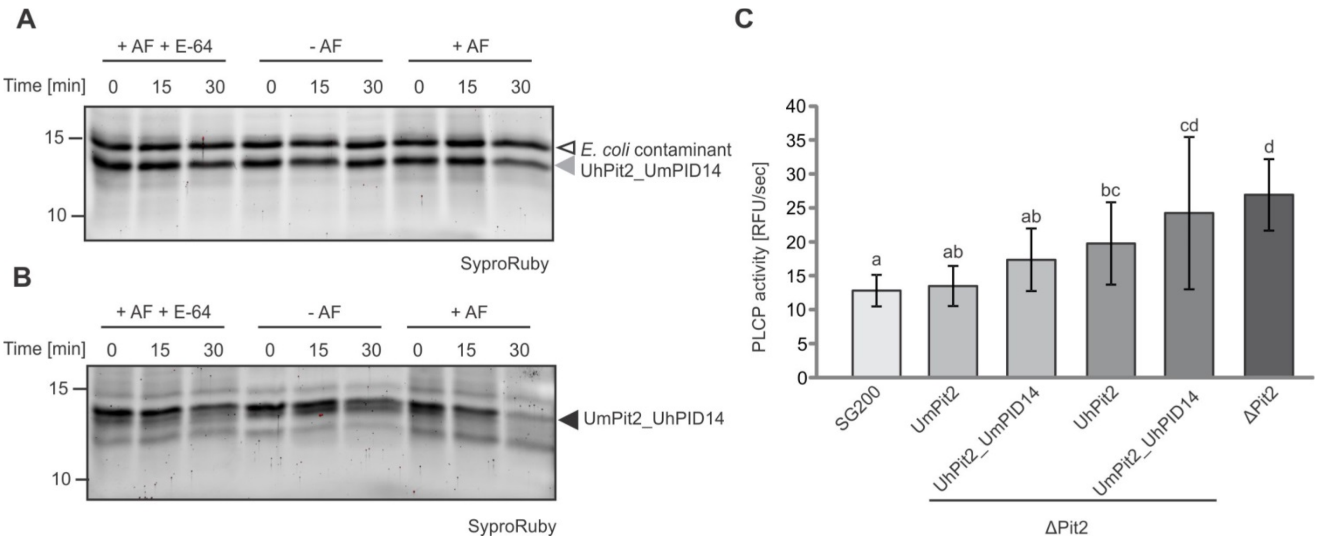

Supplementary Figure 7. Biochemical analysis of chimeras and inhibition of PLCPs during infection. (A - B) UhPit2-UmPID14 chimera is stable but not UmPit2-UhPID14. Heterologous expressed chimeras UhPit2-UmPID14 (**A**) and UmPit2-UhPID14 (**B)** were incubated for 0, 15 and 30 min with apoplastic fluid containing 20 µM E-64 (+ AF + E-64), same amount of DMSO (+ AF) or without apoplastic fluid (- AF) as a control. Reactions were stopped by adding gel loading buffer and samples were analyzed by in gel fluorescence scanning using SyproRuby staining. **(C)** Apoplastic fluids of maize seedling infected with different strains were collected 3 dpi and protein quantification was performed. Apoplastic fluids were normalized to the same concentration and PLCP activity was measured using the fluorogenic substrate Z-Phe-Arg-AMC. Lowest PLCP activity was found for SG200 and Δpit2 complemented with UmPit2. The chimera UhPit2-UmPID14 can better inhibit PLCP activity than chimera UmPit2-UhPID14. Highest PLCP activity was observed for the Δpit2 deletion strain. Values are means of three independent biological experiments and error bars represent standard deviation (SD). Letters above error bars indicate significant differences between samples (α=0.05, tukey test).

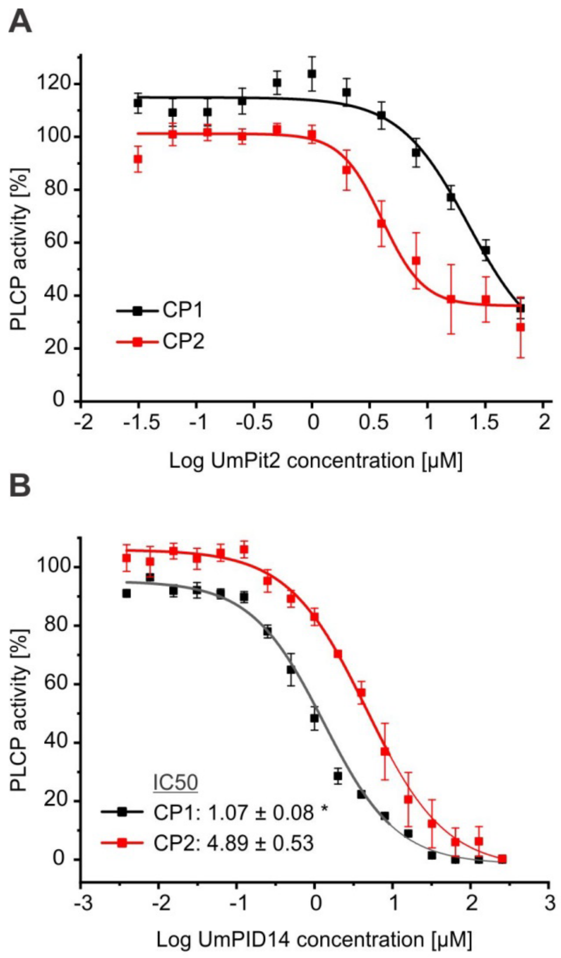

Supplementary Figure 8. Inhibitory profile of *N. benthamiana* over- expressed CP1 and CP2. Maize CP1 (CP1a) and CP2 proteases were overexpressed in *N. benthamiana* leaves via agrobacterium mediated transformation. Apoplastic fluids were isolated and tested for PLCP activity using the probe MV201 [1]. Activity was normalized based on activity blots. Apoplastic fluids containing active CP1 and CP2 were pre-incubated for 5 min with increasing concentrations of heterologous expressed UmPit2 (0 – 64 µM) **(A)** or UmPID14 (0 – 256 µM) **(B)** and PLCP activity was monitored using the fluorogenic substrate Z-Leu-Arg-AMC. PLCP activity was normalized using the non - inhibitor control and set to 100 %. Values are means of at least three independent biological replicates and the error bars represent the standard error of the mean (SEM).

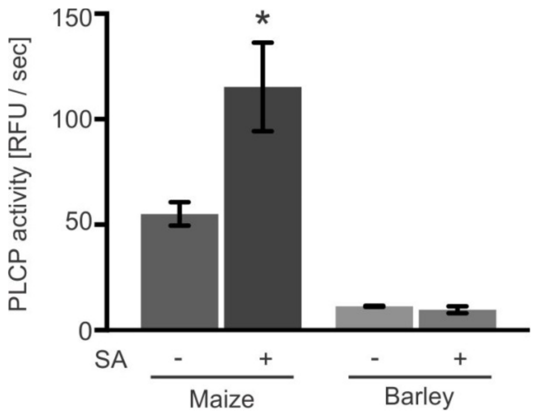

Supplementary Figure 9. SA responses in barley are different than in maize. (**A**) SA activates PLCPs in maize apoplastic fluids but not in barley. Seedlings of maize and barley were treated with or without salicylic acid and 2 days after treatment apoplastic fluids were isolated. Apoplastic fluids were normalized to the same amount of protein and activity of PLCPs was measured using the fluorogenic substrate Z-FR-AMC. Values are means of three biological replicates and error bars represent standard deviation. Asterisk indicates significant differences between maize treatments ($p = 0.0312$) based on a t-test analysis.

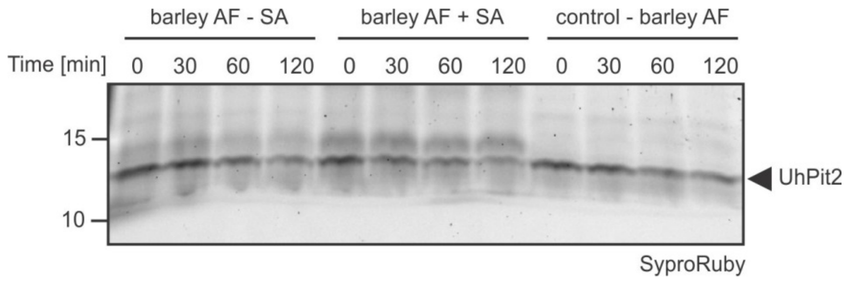

Supplementary Figure 10. UhPit2 is stable in barley apoplastic fluids. Barley seedlings were treated with or without salicylic acid and apoplastic fluids were harvested 2 days after treatment. Recombinant UhPit2 was incubated over time with barley apoplastic fluids. As a control recombinant UhPit2 without apoplastic fluids was also incubated over time to test for its stability. Samples were stained with SyproRuby and analyzed by in gel fluorescent scanning.

P_hubeiensis_SY62_Pit2

MLFRSELLLL IAAVLSISSA LHAQAAQIPL IRRSLSPSPA PAASKLDRRW WFGWSASLGK

EPDDARVGVH YIPPHWIFDH PPADSDAFAR WLARLRQQQP DFIQVTLP

Supplementary Figure 11. *Pseudozyma hubeiensis* putative Pit2 sequence. A new sequence analysis of *P. hubeiensis* using a manual search for possible open reading frames (ORF) results in an alternative gene model for a putative Pit2 homologue containing the cMIP motif (red label). SignalP 4.1 server predicts a signal peptide (blue label) for this candidate homologue.

Supplementary References

1 Richau, K. H. *et al.* Subclassification and biochemical analysis of plant papain-like cysteine proteases displays subfamily-specific characteristics. *Plant physiology* **158**, 1583-1599, doi:pp.112.194001 [pii]10.1104/pp.112.194001 (2012).

2 Mueller, A. N., Ziemann, S., Treitschke, S., Assmann, D. & Doehlemann, G. Compatibility in the Ustilago maydis-maize interaction requires inhibition of host cysteine proteases by the fungal effector Pit2. *PLoS pathogens* **9**, e1003177, doi:10.1371/journal.ppat.1003177 (2013).
