# Supplementary material for: A fungal substrate mimicking molecule suppresses plant immunity via an inter-kingdom conserved motif
Source: Nat Commun. 2019 Apr 5;10:1576. doi: 10.1038/s41467-019-09472-8 (PMC6450895; doi:10.1038/s41467-019-09472-8)
Supplement: Supplementary file 3 — Description of Additional Supplementary Files [file 41467_2019_9472_MOESM3_ESM.pdf]

Description of Additional Supplementary Files

File Name: Supplementary Data 1
Description: TOP15 proteases found in F-24 by MS analysis

File Name: Supplementary Data 2
Description: Signal peptide prediction for cMIP-containing sequences described in Figure 7

File Name: Supplementary Data 3
Description: Strains used in this study

File Name: Supplementary Data 4
Description: Primers used in this study
