# Supplementary material for: A fungal substrate mimicking molecule suppresses plant immunity via an inter-kingdom conserved motif
Source: Nat Commun. 2019 Apr 5;10:1576. doi: 10.1038/s41467-019-09472-8 (PMC6450895; doi:10.1038/s41467-019-09472-8)
Supplement: Supplementary file 4 — Supplementary Data 1 [file 41467_2019_9472_MOESM4_ESM.pdf]

Table S1. TOP15 proteases found in F-24 by MS analysis

TOP 15	UniProt ID	Description	Uniq. pept.
1	C0P8L3	Carboxypeptidase	12
**2**	**A0A1D6JG12**	**Cysteine protease 1, CP1B***	**7**
**3**	**A0A1D6DW35**	**Insect resistance 3, CP1A**	**4**
4	C0P7P2	Carboxypeptidase	4
5	A0A1D6KWW2	Subtilisin-like protease	13
**6**	**Q10717**	**Cysteine proteinase 2, CP2**	**3**
**7**	A0A1D6F2R4	Carboxypeptidase	4
**8**	**B6TIC7**	**Cysteine protease 5, XCP2**	**5**
**9**	B4F9F5	SPG31-like peptidase; Senescence-specific cysteine protease SAG12	2
**10**	B6SYG2	Carboxypeptidase;Serine carboxypeptidase-like 51	4
**11**	B6TXF8	Serine carboxypeptidase 1	2
**12**	A0A1D6FPN2	Putative subtilase family protein	3
**13**	A0A1D6HP95	Carboxyl-terminal-processing peptidase 2 chloroplastic	4
**14**	A0A1D6GGX8	Cysteine protease 1, SPG31-like peptidase	2
**15**	B4FZ79	Senescence-specific cysteine protease SAG12	2

*PLCPs are labelled red. PLCPs previously found in van der Linde et al (2012a) are shown in bold.
