# Supplementary material for: A fungal substrate mimicking molecule suppresses plant immunity via an inter-kingdom conserved motif
Source: Nat Commun. 2019 Apr 5;10:1576. doi: 10.1038/s41467-019-09472-8 (PMC6450895; doi:10.1038/s41467-019-09472-8)
Supplement: Supplementary file 5 — Supplementary Data 2 [file 41467_2019_9472_MOESM5_ESM.pdf]

Table S2. Signal peptide prediction for cMIP-containing sequences described in Figure 7

Species name	Classification	Sequence name	length	SignalP 2.0 Results									TM-HMM 2.0 Results				TargetP 1.1 Results					
				For Eukaryotes			For Gram -ve bacteria			For Gram +ve bacteria												
				Secreted?	Signal peptide probability	Cleavage site	Secreted?	Signal peptide probability	Cleavage site	Secreted?	Signal peptide probability	Cleavage site	ExpAA	First60	PredHel	Topology	mTP probability	SP probability	other	Localisation	Reliability Class	Signal length
Amycolatopsis mediterranei	Gram+ bacterium	Amed	197	No	0.007	0	No	0	0	No	0.006	0	62.14	0	3	i115-137o142-164i177-196o	0.092	0.063	0.879	_	2	-
Burkholderia vietnamiensis G4	Gram- bacterium	Bviet	604	No	0.042	0	No	0.003	0	No	0.001	21	4.03	1.52	0	o	0.449	0.194	0.244	M	4	53
Microbacterium	Gram+ bacterium	Micrbo	195	No	0.004	57	No	0	66	No	0.03	66	96.39	20.89	4	o40-62i75-97o117-151i158-180o	0.109	0.348	0.673	_	4	-
Streptomyces sp	Gram+ bacterium	Strep	140	No	0	68	No	0	0	No	0	0	61.05	2.47	3	i58-80o85-107i120-139o	0.049	0.245	0.889	_	2	-
Micobacterium	Gram+ bacterium	Mycob	294	No	0.864	25	No	0.868	69	No	0.38	27	183.52	26.1	9	o4-21i52-70o75-97i104-126o136-158i165-182o192-214i226-248o258-277i	0.018	0.926	0.116	S	1	94
Pseudozyma antarctica	Ustilaginomycetous yeast	Pant	137	No	0.982	51	No	0.737	52	No	0.486	52	21.63	21.63	1	o28-50i	0.494	0.169	0.374	M	5	114
Pseudozyma hubeiensis	Ustilaginomycetous yeast	Phub	150	No	0	0	No	0	66	No	0	66	0	0	0	o	0.236	0.048	0.796	_	3	-
Sporisorium reilianum	Smut fungus	Srei	117	YES	0.999	23	YES	0.998	23	YES	0.966	23	17.15	17.15	1	i7-24o	0.201	0.942	0.007	S	2	23
Ustilago hordei	Smut fungus	Uhor	120	YES	0.983	24	No	0.861	17	No	0.773	22	2.94	2.94	0	o	0.389	0.819	0.009	S	3	24
Ustilago maydis	Smut fungus	Umay	118	YES	0.999	25	YES	0.999	17	YES	1	25	15.48	15.48	0	o	0.178	0.843	0.011	S	2	25
Melanopsichium pennsylvanicum	Smut fungus	Mpen	109	YES	1	24	YES	1	24	YES	1	24	4.95	4.95	0	o	0.239	0.879	0.011	S	2	24

ExpAA = The expected number of amino acids intransmembrane helices. If this number is larger than 18 it is very likely to be a transmembrane protein
First60 = The expected number of amino acids in transmembrane helices in the first 60 amino acids of the protein. If this number more than a few, you should be warned that a predicted transmembrane helix in the N-term could be a signal peptide.
PredHel = The number of predicted transmembrane helices.
Topology = The topology is given as the position of the transmembrane helices separated by 'i' if the loop is on the inside or 'o' if it is on the outside. The above example 'i7-29o44-66i87-109o' means that it starts on the inside, has a predicted TMH at position 7 to 29, the outside, then a TMH at position 44-66 etc.

1. Amed_Pit2	*Amycolatopsis mediterranei*	Bacterium	Gram-positive produces Rifampicin
2. Micrbo_Pit2	*Microbacterium* sp. 292MF	Bacterium	Gram-positive Actinobacteria
3. Mycob_Pit2	*Mycobacterium tusciae*	Bacterium	Gram positive
4. Strep_Pit2	*Streptomyces* sp	Bacterium	Gram-positive Actinobacteria
5. Bviet_Pit2	*Burkholderia vietnamiensis* G4	Bacterium	Gram-negative, Proteobacteria
6. Mpen_Pit2	*Melanopsichium pennsylvanicu*	Smut Fungus	
7. Umay_Pit	*Ustilago maydis*	Smut Fungus	
8. Pant_Pit2	*Pseudozyma antarctica*	Ustilaginomycetous yeast produces lipids	
9. Phub_Pit2	*Pseudozyma hubeiensis*	Ustilaginomycetous yeast produces lipids	
10. Srei_Pit2	*Sporisorium reilianum*	Smut Fungus causes disease on Sorghum	
11. Uhor_Pit2	*Ustilago hordei*	Smut Fungus causes barley Covered smut	
