# Supplementary material for: A fungal substrate mimicking molecule suppresses plant immunity via an inter-kingdom conserved motif
Source: Nat Commun. 2019 Apr 5;10:1576. doi: 10.1038/s41467-019-09472-8 (PMC6450895; doi:10.1038/s41467-019-09472-8)
Supplement: Supplementary file 6 — Supplementary Data 3 [file 41467_2019_9472_MOESM6_ESM.pdf]

Table S3. Strains used in this study

strains used — Phleomycin (P), Hygromycin (H), Carboxin (C), Carbenicillin (Carb), Rifampicin (R), Gentamicin (G)

strain	genotype	resistance	reference	comments
SG200	a1mfa2bW2 bE1	P	Kamper et al., 2006	
SG200Δpit2	a1mfa bW2bE1 Δum01375::hph	P, H	Doehlemann et al., 2011	
SG200Δpit2-UhPit2	a1mfa2 bW2bE1 Δum01375::hph ipr[Ppit2::uh02064]ips	P, H, C	this work	without introns
SG200Δpit2-SrPit2	a1mfa2 bW2bE1 Δum01375::hph ipr[Ppit2::sr10529]ips	P, H, C	this work	without introns
SG200Δpit2-MpPit2	a1mfa2 bW2bE1 Δum01375::hph ipr[Ppit2::mp4_3204_1]ips	P, H, C	this work	without introns
SG200Δpit2-UmPID14	a1mfa2 bW2bE1 Δum01375::hph ipr[Ppit2-UmPID14]ips	P, H, C	this work	without introns
SG200Δpit2-UhPID14	a1mfa2 bW2bE1 Δum01375::hph ipr[Ppit2-UhPID14]ips	P, H, C	this work	without introns
SG200Δpit2-UmPit2-UhPID14	a1mfa2 bW2bE1 Δum01375::hph ipr[Ppit2::um01375UhPID14]ips	P, H, C	this work	without introns
SG200Δpit2-UhPit2-UmPID14	a1mfa2 bW2bE1 Δum01375::hph ipr[Ppit2::uh02064UmPID14]ips	P, H, C	this work	without introns
SG200Δpit2-UmPit2	a1mfa2 bW2bE1 Δum01375::hph ipr[Ppit2::um01375]ips	P, H, C	this work	without introns
SG200Δpit2-UmPit2_RW mut	a1mfa2 bW2bE1 Δum01375::hph ipr[Ppit2::um01375 R51AW52A mut]ips	P, H, C	this work	without introns
SG200-UmPit2-mCherry-HA	a1mfa2bW2 bE1 SG200 ipr[Ppit2::um01375-mCherry-HA]ips	P, C	this work	
SG200-SP-mCherry-HA	a1mfa2bW2 bE1 SG200 ipr[Ppit2::Pit2SP-mCherry-HA]ips	P, C	this work	
SG200-mCherry	a1mfa2bW2 bE1 SG200 ipr[Ppit2::mcherry]ips	P, C	this work	
SG200-UhPit2-mCherry-HA	a1mfa2 bW2bE1 SG200 ipr[Ppit2::uh02064-mCherry-HA]ips	P, C	this work	without introns
SG200-SrPit2-mCherry-HA	a1mfa2 bW2bE1 SG200 ipr[Ppit2::sr10529-mCherry-HA]ips	P, C	this work	without introns
SG200-MpPit2-mCherry-HA	a1mfa2 bW2bE1 SG200 ipr[Ppit2::mp4_3204_1-mCherry-HA]ips	P, C	this work	without introns
SG200-UmPit2-UhPID14-mCherry-HA	a1mfa2bW2 bE1 SG200 ipr[Ppit2::um01375UhPID14-mCherry-HA]ips	P, C	this work	without introns
SG200-UhPit2-UmPID14-mCherry-HA	a1mfa2bW2 bE1 SG200 ipr[Ppit2::uh02064UmPID14-mCherry-HA]ips	P, C	this work	without introns
SG200-UmPID14-mCherry	a1mfa2bW2 bE1 SG200 ipr[Ppit2-UmPID14-mcherry]ips	P, C	this work	
SG200-UhPID14-mCherry	a1mfa2bW2 bE1 SG200 ipr[Ppit2-UhPID14-mcherry]ips	P, C	this work	
SG200-UmPit2_RW mut-mCherry	a1mfa2bW2 bE1 SG200 ipr[Ppit2::um01375 R51AW52A mut-mCherry]ips	P, C	this work	without introns
GV3101_CP1a	pL1M-F1-CP1A_nogran-Streptwin::2x35S	Carb, R, G	this work	for *N. benthamiana* expression
GV3101_CP2	pL1M-F1-CP2-Streptwin::2x35S	Carb, R, G	this work	for *N. benthamiana* expression
GV3101_p19	pL1M-F3_p19::2x35S	Carb, R, G	this work	for *N. benthamiana* expression
