# Supplementary material for: A fungal substrate mimicking molecule suppresses plant immunity via an inter-kingdom conserved motif
Source: Nat Commun. 2019 Apr 5;10:1576. doi: 10.1038/s41467-019-09472-8 (PMC6450895; doi:10.1038/s41467-019-09472-8)
Supplement: Supplementary file 7 — Supplementary Data 4 [file 41467_2019_9472_MOESM7_ESM.pdf]

Table S4. Primers used in this study

primers used

name	sequence 5' - 3'	use
Tnos_fw	ATACCCGGGCGGCCGCTAGCTCTAGAGCCGCCCGGCTGCAGATCGT	assembly of p123_Ppit2
Pit2_SP_fw	ATACCGCGGATGCTGTTTCGCTCAGCCTTTGTTCTGCTCATCGTGGCCTTTGCAAGTGCATGCCTGGTGCAACATGTTCAAGCTATTCCGCCCGGGATA	cloning of Um01375 signal peptide
Pit2_SP_rv	TATCCCGGGCGGAATAGCTTGAACATGTTGCACCAGGCATGCACTTGCAAAGGCCACGATGAGCAGAACAAAGGCTGAGCGAAACAGCATCCGCGGTAT	cloning of Um01375 signal peptide
UmPit2_Xma_fw	ATACCCGGGATTCCGGTGCGTCGATCGCTC	amplification of Um01375 without signal peptide
UmPit2_Xba_rv	GCGTCTAGATTATTCCCAGATGACCACATCTCCG	amplification of Um01375 without signal peptide
UhPit2_Xma_fw	TGTCCCGGGAACAGACCTTTACGCAGAGC	amplification of Uh02064 without signal peptide
UhPit2_Xba_rv	CACTCTAGACTAAGACTTCTTGCTGCTGC	amplification of Uh02064 without signal peptide
SrPit2_Xma_fw	TATCCCGGGATGCCAGCCATGCGTCGATC	amplification of sr10529 without signal peptide
SrPit2_Xba_rv	CACTCTAGATCAGTGGCTCTTGTACCCAA	amplification of sr10529 without signal peptide
MpPit2_Xma_fw	TATCCCGGGGGCCGAATCCAAGCTGTACC	amplification of mp4_3204_1 without signal peptide
MpPit2_Xba_rv1	TGTTCTAGATTACTGCGAAAAAGTGACCC	amplification of mp4_3204_1 without signal peptide
UhPID14_fw	AGAGCCCGGGATGAAGCTCCACCGACGATGGTACTTTCTCTGGCCCGGCTCACTTTAATCTAGAATAT	cloning of UhPID14
UhPID14_rv	ATATTCTAGATTAAAGTGAGCCGGGCCAGAGAAAGTACCATCGTCGGTGGAGCTTCATCCCGGGCTCT	cloning of UhPID14
UmPID14_fw	AGAGCCCGGGATGAAGCTCAACCGGAGATGGTGGTTCGGCTTCACAGGTTCGCTCTAATCTAGAATAT	cloning of UmPID14
UmPID14rv1	ATATTCTAGATTAGAGCGAACCTGTGAAGCCGAACCACCATCTCCGGTTGAGCTTCATCCCGGGCTCT	cloning of UmPID14
UmPit2-UhPID14_fw	AAGCTCCACCGACGATGGTACTTTCTCTGGCCCGGCTCACTTGGCAAGGAACCTGACAACGGCCAAGTACAG	insertion of UhPID14 into Um01375
UmPit2-UhPID14_rv	AAGTGAGCCGGGCCAGAGAAAGTACCATCGTCGGTGGAGCTTGCCAGCAGCCGAGCTCATTGAGGCATCGG	insertion of UhPID14 into Um01375
UhPit2-UmPID14_fw	AAGCTCAACCGGAGATGGTGGTTCGGCTTCACAGGTTCGCTCGCTCCCAAACCTCCACGTGA	insertion of UmPID14 into Uh02064
UhPit2-UmPID14_rv	GAGCGAACCTGTGAAGCCGAACCACCATCTCCGGTTGAGCTTGGTGATGTAATTGTCGTTGT	insertion of UmPID14 into Uh02064
UmPit2_RW_mut	GGCAAGCTCAACCGGGGAGGGTGGTTCGGCTTCACAGG	Um01375 site directed mutagenesis R48G-W49G
CP1A-F-BsaI	GGTCTCAAATGGCTGCCTCCACCACG	For direct cloning into pICH47732 binary vector
CP1A_nogranulin_R_BsaI	GGTCTCACGAACCGTTAGCGCCCTCCTTCAA	For direct cloning into pICH47732 binary vector
CP2-F-BsaI	GGTCTCAAATGGCCCCACGCCGCCTG	For direct cloning into pICH47732 binary vector
CP2-R-BsaI	GGTCTCACGAACCTGCGACAATAGGGTAGGA	For direct cloning into pICH47732 binary vector
